# Person-Centred Care Including Deprescribing for Older People

**DOI:** 10.3390/pharmacy7030101

**Published:** 2019-07-25

**Authors:** Heather Smith, Karen Miller, Nina Barnett, Lelly Oboh, Emyr Jones, Carmel Darcy, Hilary McKee, Jayne Agnew, Paula Crawford

**Affiliations:** 1 Leeds Teaching Hospitals NHS Trust, Leeds LS9 7TF, UK; 2 South Eastern Health and Social Care Trust, Belfast BT16 1RH, UK; 3 London North West University Healthcare NHS Trust, London, HA1 3UJ, UK; 4 Medicines Use and Safety Division, NHS Specialist Pharmacy Service, London, HA1 3UJ, UK; 5 Guys and St Thomas NHS Trust (Community Health Services), London SE1 7EH, UK; 6 Cardiff and Vale University Health Board, Cardiff CF14 4XW, UK; 7 Western Health and Social Care Trust, Londonderry BT47 6SB, UK; 8 Northern Health and Social Care Trust, Antrim BT41 2RL, UK; 9 Southern Health and Social Care Trust, Portadown BT63 5QQ, UK; 10 Belfast Health and Social Care Trust, Belfast BT9 7AB, UK

**Keywords:** deprescribing, elderly, medicines, multimorbidity, polypharmacy

## Abstract

There is concern internationally that many older people are using an inappropriate number of medicines, and that complex combinations of medicines may cause more harm than good. This article discusses how person-centred medicines optimisation for older people can be conducted in clinical practice, including the process of deprescribing. The evidence supports that if clinicians actively include people in decision making, it leads to better outcomes. We share techniques, frameworks, and tools that can be used to deprescribe safely whilst placing the person’s views, values, and beliefs about their medicines at the heart of any deprescribing discussions. This includes the person-centred approach to deprescribing (seven steps), which incorporates the identification of the person’s priorities and the clinician’s priorities in relation to treatment with medication and promotes shared decision making, agreed goals, good communication, and follow up. The authors believe that delivering deprescribing consultations in this manner is effective, as the person is integral to the deprescribing decision-making process, and we illustrate how this approach can be applied in real-life case studies.

## 1. Introduction

Polypharmacy is the use of multiple medicines, and is increasing worldwide due to an ageing population, advances in the treatment of disease, and the increasing prevalence of multimorbidity [1,2], i.e., the presence of two or more long-term conditions (LTCs) [3]. Other factors may also be at play such as single condition clinical guidelines, care pathways, and even processes for clinical remuneration [1,4,5]. Clinical guidelines are usually for single conditions, and the evidence base for these is taken from clinical trials using younger people, where people with multimorbidity and polypharmacy are routinely excluded [1,3,6]. As a consequence, the evidence base for treating multiple LTCs with polypharmacy is poor [1]. Patients with multimorbidity and a limited life expectancy or severe frailty may obtain limited benefit from medicines treating a single condition [3], especially secondary prevention medicines. Polypharmacy is common in care homes or long-term care facilities where residents tend to be older and are more likely to have multimorbidity than people living in domiciliary settings [7]. There is concern internationally that many older people are using an inappropriate number of medicines and that complex combinations of medicines may cause more harm than good. Therefore, providing optimised care for older people with multimorbidity and polypharmacy is an issue that requires international attention [5].

The Kings Fund [1] has proposed the use of the terms ‘appropriate’ and ‘problematic’ polypharmacy. Appropriate polypharmacy has been defined as prescribing for an individual for complex conditions or multiple conditions in circumstances where medicines use has been optimised and where the medicines are prescribed according to best evidence. Appropriate polypharmacy can extend life expectancy and quality of life. Problematic polypharmacy has been defined as the prescribing of multiple medications inappropriately or where the intended benefit of the medication is not realised [1]. Problematic polypharmacy has been linked to a wide range of negative consequences including increased adverse drug reactions (ADRs), drug–drug and drug–disease interactions [1,5,8], acute renal failure, cognitive impairment [5], falls [4,5,9,10], frailty, sarcopenia [5], healthcare costs [8], hospital admissions [5,11], mortality [5,12], reduced adherence to medicines [1,8], less functional capacity [5,8], and a decline in quality of life [1]. However, it can be difficult to determine whether negative consequences are due to polypharmacy or the underlying conditions for which the medicines have been prescribed [5]. 

Deprescribing is defined as the process of tapering, stopping, discontinuing or withdrawing drugs, with the goal of managing polypharmacy and improving outcomes. Deprescribing reduces the number of medicines prescribed, adverse drug reactions, and medicine costs, but there is little evidence of impact on clinical outcomes with the exception of falls [2,6,12]. Reassuringly, available evidence does suggest that deprescribing is safe [2].

There is little evidence to guide the person and/or the prescriber in how to reach a decision on what medicines to stop, and how to deprescribe in practice [4,13,14]. Effective deprescribing is a complex process [15] with a number of components including participation by patients and/or relatives and carers [16,17], prescriber/patient relationships and communication [18,19,20], and shared decision making [17]. 

Appropriate polypharmacy requires individualised, patient-centred care using medicines optimisation [4]. Medicines optimisation is a process that allows patients to gain the most net benefit from taking medication where medicines are prescribed taking the patient’s preferences and experiences into account [21] and acknowledging that what is most valued by the person might be different to what is most valued by the healthcare professional [15].

A clinical medication review has been defined as a structured, critical examination of a person’s medicines with the objective of reaching an agreement with the person regarding treatment, optimising the impact of medicines, minimising the number of medication-related problems, and reducing waste [22].

We will now discuss how a person-centred, clinical medication review with a focus on deprescribing can be provided in real world, clinical practice based on the clinical experience of the authors and provide examples of cases where this approach has been successfully implemented.

## 2. Person-Centred Care for Older People

The original concept of person-centred care was initially described in the late 1960s as “understanding the patient as a unique human being” [23,24]. This concept is as important as ever in a world of healthcare that has seen many changes. Historically in healthcare, the relationship between the person and clinician tended to be more of a paternalistic relationship. It was a culture of ‘clinician knows best’, and people tended to accept the recommendation of a clinician without question. The clinician had all the knowledge and the subsequent power that knowledge affords, leading to information asymmetry [25].

Times are changing, especially with the enhancement in technology, which allows people to obtain information freely and quickly through a click of a button. The rise of “Dr Google” has reduced this imbalance of knowledge. People who are interested or worried have lots of time to research and can become overloaded with information. In some instances, the person can be more conversant than the clinician, which can be a difficult concept to comprehend. The role of the clinician has to adapt with this culture change with a move towards more of a coaching relationship by supporting people to decipher complex information. There is a synergistic relationship between clinicians being the experts in the field and people being experts on their own personal needs.

The evidence supports that if clinicians actively include people in decision making, it leads to better outcomes [26]. This helps improve engagement, thus inspiring people to take more accountability for their health and well-being, whilst empowering them to manage their own condition. The increased accountability of shared decisions can lead to more positive behaviours including better adherence to regimens and more educated lifestyle choices. This results in better personal experience and improved consultation satisfaction, and importantly a more knowledgeable and informed person.

Person-centred care is not ‘what the person says, goes’. Clinicians should promote person autonomy, but it does not mean ignoring important issues such as research evidence, cost effectiveness, and value-based care. As with everything in healthcare, adopting the right balance is crucial.

### 2.1. How to Provide Person-Centred Care (PCC)

A key component of person-centred care is the development of a positive relationship between the clinician and the person that will help improve rapport and build trust. Improving communication leads to a more meaningful conversation and productive consultation. By investing time in getting to know the person, the clinician can get an essential insight into what is important to them, eliminating preconceived conceptions. What is important to the clinician does not necessarily indicate it is important to the person. There needs to be a shift in focus from “what is wrong with you” to “what matters to you”. Risks that are unacceptable to a clinician may actually be satisfactory to the person.

As active partners, the person and clinician should prioritise care based on the preferences and goals of the person. This is the basis of shared decision making. In order for effective shared decision making to occur, a clinician needs to see the person as the individual, not just as a condition. Clinicians need to show flexibility within guidelines to take individual preferences into account, focusing on quality of life. After all, they are meant to be *guide*lines, not *tram*lines. It is also essential to consider the needs of the person over the needs of the service, which is not necessarily easy in the current climate.

In the grand scheme of things, taking medication may not be on top of a person’s life agenda. Clinicians need to support people to ensure medications fit around their lifestyle, not vice versa.

### 2.2. What Is the Role of the Clinician

The clinician must promote an open environment that is conducive to allow person-centred care to occur. To permit this, the clinician will discuss all the possible options whilst explaining the associated risk and benefits of each, ensuring that the information is tailored to the level that the person understands. By obtaining the person’s perspective, the clinician and person can explore all the options to find the most suitable solution that reflects specific goals, values, and preferences. Providing the person is fully informed and has capacity, clinicians are required to be non-judgemental and support the person’s preference, without reference to the clinicians’ personal opinion of the person’s choice. Person-centred care is a dynamic process. People can often change their mind after a period of reflection. Clinicians should periodically reassess the person’s preferences and goals over time through ongoing discussions, not just as a one-off review.

### 2.3. How Can We Apply Person-Centred Care (PCC) to Deprescribing

**Figure 1:**
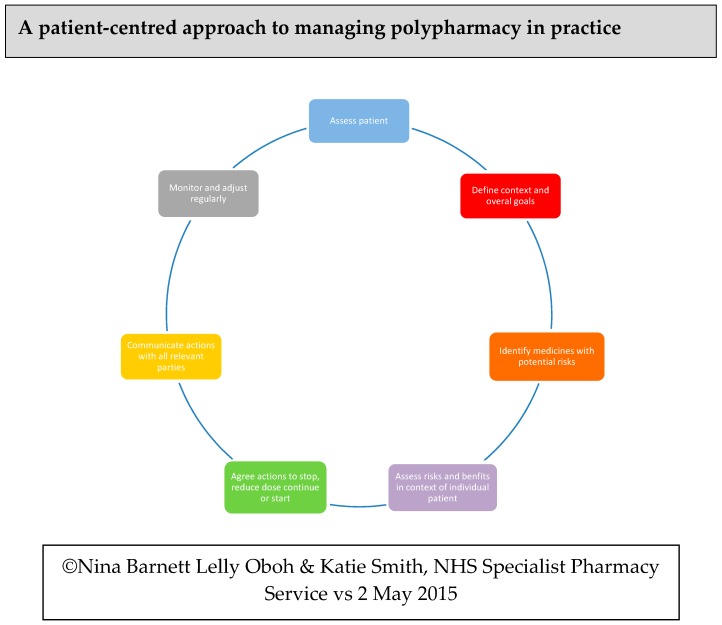
A patient-centred approach to deprescribing (seven steps).

Barnett et al. (2015) described a person-centred approach to managing polypharmacy and deprescribing through seven steps [27]. This approach incorporates the identification of the person’s priorities and the clinician’s priorities in relation to treatment with medication and promotes shared decision making and agreed goals as well as good communication and follow up, as illustrated below in Figure 1.

In order to optimise the effectiveness of a consultation, it is important that the clinician has prepared sufficiently in advance. This could include interrogating the medical notes to produce a rough plan and identify key areas to discuss with the person. This will include reviewing the medication list and ensuring that all the biochemistry is current. It is helpful to align each medication to a recorded diagnosis in order to highlight medication that may need further investigation. Colleagues from another sector may need to be contacted to determine why a particular medicine has been prescribed. A review prior to the face-to-face medication review can help highlight potentially inappropriate prescriptions that the clinician may wish to discuss with the person. Tools are available to support effective medication review, which can be utilised for medication appropriateness evaluation, such as for example STOPP START [28], or the Beers criteria [29,30]. Both of these tools list medications that may be inappropriate for older people, with the former created for the United Kingdom (UK) and Ireland, and the latter focusing on medications available in the United States (USA).

Some practitioners find it helpful to divide the medication under review into two main classes: those that are for day-to-day symptom control, and those that are for managing risk reduction, as different questions may need to be asked for the two groups. The authors propose a simple approach to medication review, which identifies the rationale for treatment and questions to consider before prescribing, as shown in Table 1 below. Where it is unclear whether medication is controlling symptoms, reduction or withdrawal of medication may be undertaken, as appropriate to the condition being treated and the medication in question. This would be followed by reassessment at specified intervals and recommencement if required. Where medication for risk reduction has been prescribed, a discussion with the person would include what the person wants to achieve through medication taking, considering the benefits, burden, and risk from their perspective. The clinician would contribute an evaluation by the clinician of the clinical benefits and risks. These factors would be evaluated together to reach a shared decision between the person and clinician regarding the next steps.

**Figure 2:**
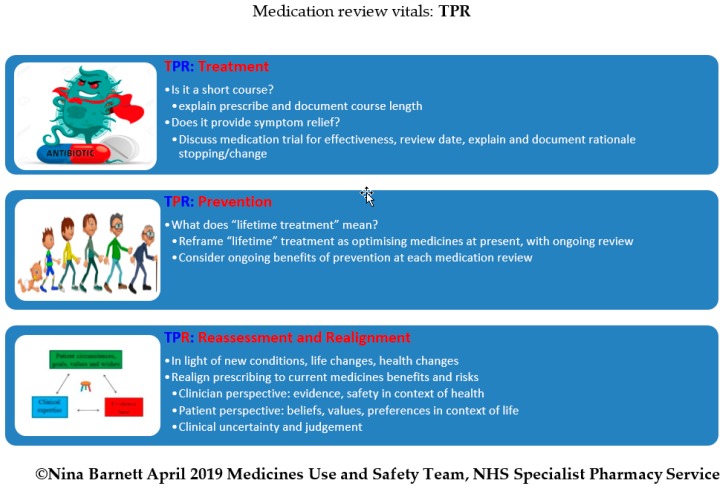
The medication review vitals: TPR (Treatment, Prevention, Reassessment).

The ‘TPR’ (Treatment, Prevention, Reassessment) tool is another useful framework that expands on the concept of rationale for treatment, in order to help focus on the key parts of a medication review, helping simplify and maximise the effectiveness of the consultation. This is illustrated in Figure 2 below.

Although it is helpful to prepare for the consultation in advance, the real work starts when the person is present. People often have their own view of their medicines and their own goals, which may differ from the clinician’s view, so it is imperative to understand what’s important to them. What are their actual goals from taking medication, and do they know why they are taking them? Do they have any concerns or particular worries? By assessing their understanding of the condition and medication, it allows the clinician to pitch the consultation at the correct level.

It is helpful to begin with broad open questions initially to gather as much information as possible before moving onto more closed questions for specific information.

Using a health coaching model—such as “the four Es” [31], shown below in Table 2—is a useful way of allowing clinicians to use their expertise to educate the person with the aim of altering their health behaviours, in a way that is aligned with what they want to know about medicines. It also includes methods of helping patients take ownership for their medicines-related activities and supports embedding these changes into everyday behaviours.

It is of the utmost importance that the clinician assesses the person’s adherence through cautious questioning without being seen to apportion blame. Using non-judgemental language and looking for clues such as prescribing/dispensing dates and patterns can be useful to identify non-adherence. Applying a framework such as the COM-B framework [32], which is a behavioural framework that can be applied to medication adherence, can aid the clinician and person to identify any barriers to adherence, as shown in Table 3 below.

Once the clinician and person have a rapport and a common purpose has been established, the next step is to work out a plan together. The focus should be on the aspects that are most important to the person. The clinician should also attempt to address medicines that either carry the highest risk or the lowest potential for benefit. Clinicians need to ensure that if a medicine is stopped or reduced, the person knows what signs and symptoms should prompt them to seek further advice. This provides a safety net if there are adverse consequences of stopping medicines, and empowers the person to manage their own condition. Once a plan has been agreed by both parties, this should then be documented in detail in the clinical notes or records and shared with all key individuals. If using a prescribing system, then it is good practice to add flags/notes to highlight the plan to others in the event of any queries.

Finally, the intervention should be monitored and reviewed. Once the deprescribing process is complete, the person and clinician can move on to the next potentially inappropriate prescription and repeat the same method.

It is important to appreciate that deprescribing is not a straightforward task, which may explain why it often gets overlooked. Effective communication regarding potential risks and benefits is essential if the person is to be able to participate fully in the process. A lack of time is often highlighted as a barrier to deprescribing. It is true that tackling difficult issues may require more frequent visits initially, but focus needs to be on “the bigger picture” and the potential long-term health benefits. Deprescribing can be done in manageable chunks by reviewing one medication at a time and using every interaction with a person as an opportunity to review medications.

One of the biggest challenges is reviewing a medication that was started by another clinician. Unfortunately, professional silos still exist in healthcare with a lack of clear documentation being a major obstacle. Having multiple prescribers exacerbates the problem. However, focus needs to be on the holistic needs of the person and on quality of life. The habitual “if it isn’t broke, don’t fix it” mentality to a medication review needs to be challenged. Clinicians need to be able to review the medicines and be confident that each one passes the common sense question: “Does the benefit of this medicine outweigh the potential harm for the individual person?”

By following a systematic person-centric process, deprescribing is possible, and can be done safely.

## 3. Deprescribing Case Studies

Detailed below are two case studies that demonstrate the person-centred approach to deprescribing (seven steps) shown in Figure 1 in practice. Ethics approval was not required, as these cases are anonymised and were undertaken during the course of usual clinical practice.

### 3.1. Intermediate Care

#### 3.1.1. Steps 1 and 2: Assess Patient; Define Context and Overall Goals

Mrs. AT, an 89-year-old lady, was admitted to intermediate care from an acute hospital following treatment for a UTI (urinary tract infection) and increased knee pain.

She had a history of osteoporosis, ischaemic heart disease, mild aortic regurgitation, diverticular disease, gallstones, restless legs syndrome, recurrent UTIs, hypertension, hyperlipidaemia, osteoarthritis in her right hip and knee resulting in chronic right hip pain, a history of falls, a previous fractured humerus following a fall, and heartburn and reflux.

Her weight was 74 kgs, height 160 cm, and she had no renal impairment of note. She was considered to be mildly frail, using the Clinical Frailty Scale (Rockwood), with a score of 4–5. She was prescribed 13 drugs. The potential of harm from polypharmacy is known to be greater in frail patients [15].

She lived in a two-storey house with her husband and was independent in personal care. She was independent in the day-to-day management of her medicines with support from her daughter who completed a weekly monitored dosage system (MDS).

The intermediate care pharmacist met with Mrs. AT to get to know her and find out her perspective on her medicines. She was stoic and initially did not feel that she had any issues or concerns with her medication, but with further discussion, she admitted to having difficulty swallowing her Forceval^®^ capsules (multivitamin and mineral supplement), and that she would like to reduce the number of medicines she currently took.

She had huge confidence in her general practitioner (Family doctor, GP) and was happy to follow his instruction, but she was pleasantly surprised when she realised that stopping some of her medicines could be an option. The recommendation of a patient’s GP has been described as a strong influence towards, or against deprescribing. Patient perspective is a known barrier to deprescribing. Anderson et al. have detailed barriers and enablers to deprescribing [33].

Her medication was as follows:

- Nitrofurantoin 50 mg at night
- Lansoprazole 30 mg twice a day
- Perindopril 4 mg in the morning
- Furosemide 40 mg in the morning, 20 mg at lunchtime
- Pregabalin 25 mg at night
- Bisoprolol 2.5 mg in the morning
- Carbocisteine 750 mg twice a day
- Amitriptyline 10 mg at night
- Aspirin 75 mg in the morning
- Ropinirole 1 mg at night
- Montelukast 10 mg at night
- Clenil modulate 100 mcg 1 puff twice a day
- Salbutamol 100 mcg inhaler 1 to 2 puffs when required
- Paracetamol 1 g 4-6 hourly when required
- Forceval one in the morning
- Buprenorphine patch 10 mcg once a week on a Tuesday (this had been increased from 5 mcg during the hospital admission)
- Ispaghula husk one sachet twice a day
- Lidocaine 5% patch 1 once a day to both knees

She had no allergies.

The anticholinergic effect on cognition (AEC) score was 3, and the anticholinergic burden (ACB) score was 4 for all her medication [34,35].

#### 3.1.2. Steps 3 and 4: Identify Medicines with Potential Risks; Assess Risks and Benefits in Context of Individual Patient

The pharmacist had undertaken a pre-person review and identified the following potentially inappropriate prescriptions to discuss with Mrs AT.

- Nitrofurantoin
- Lansoprazole
- Furosemide
- Pregabalin
- Amitriptyline
- Inhalers
- Montelukast
- Carbocisteine
- Forceval
- Lidocaine

**i.** Nitrofurantoin 50 mg at night was prescribed to prevent any urinary tract infections (UTI). Antibiotics are given in this way to allow a period of bladder healing which makes a UTI much less likely. There is no evidence that they have any additional benefit beyond 6 to 12 months of treatment. Treatment should be discontinued ideally after 6 months. Mrs. AT had been taking the nitrofurantoin for 6 months. She was frightened that if she stopped it, the UTIs would come back. Her GP had prescribed it for her, and since starting it, she had had no UTIs. It was discussed with her GP, who was happy to continue it, as it was working, and Mrs. AT did not have any side effects. It was agreed to revisit it again with Mrs. AT in a month’s time.

**ii.** Mrs. AT had been taking lansoprazole 30 mg twice a day for the past couple of years. She had initially taken one capsule once a day, but after a bout of severe reflux, it had been increased to twice a day. The dose had since remained at 30 mg twice a day. The reflux symptoms may have been caused by some of the other medication prescribed for Mrs. AT, e.g., aspirin, amitriptyline. There is no evidence of any benefit of taking the full therapeutic dose of a PPI (proton pump inhibitor) for more than eight weeks. PPIs, especially if used in high doses and over long durations (>1 year), may increase the risk of hip, wrist, and spine fracture, predominantly in the elderly or in the presence of other recognised risk factors [36]. Mrs. AT had osteoporosis and a history of falls. She currently had no reflux symptoms. Mrs. AT agreed to a trial of reducing the dose to 15 mg twice a day, and was reassured that if her reflux symptoms returned, the dose could be increased back to 30 mg twice a day. The pharmacist monitored Mrs. AT’s symptoms. If she remained symptom-free, the next step would be to try reducing the dose to 15 mg once a day and monitor for reflux.

**iii.** Furosemide had been prescribed for breathlessness the previous year. Mrs. AT had a history of falls, and furosemide is a medication associated with falls. Mrs. AT had mild aortic regurgitation, but did she need a twice-a-day regimen? Cohen et al. found that in patients with severe heart failure, the natriuretic and diuretic effects are similar whether oral furosemide in tablet or solution form is administered in a once or twice-daily schedule [37]. Elliott et al. reported that the diuretic effect is probably governed by the total daily dosage of loop diuretic and the total amount reaching the tubular lumen, and is not significantly influenced by different schedules of dose administration [38]. Anisman et al. found that loop diuretics respond in an all-or-none fashion. A common error with loop diuretics is to prescribe multiple, different daily doses. Anisman recommended finding a dose that works, and using only that dose [39].

The GP did not agree to change the furosemide dose. The 40 mg in the morning and 20 mg at lunchtime had been prescribed by a cardiologist during a previous hospital admission. In patients with multiple co-morbidities, medications are often prescribed by multiple prescribers in different prescribing environments. This can make medication review more difficult, as the original prescriber will often not communicate their reasoning for the prescription or the expected duration of that dose to other prescribers.

**iv and v.** Mrs. AT was prescribed pregabalin and amitriptyline. She believed these to be for her hip pain. There was no documentation of neuropathic pain in Mrs. AT’s medical record, and she had no recollection of ever having had nerve pain symptoms, e.g., numbness or tingling. Both drugs had been started three years previously. The pregabalin dose of 25 mg at night was sub-therapeutic. Amitriptyline causes sedation, and is a medication that is implicated with falls. It has anticholinergic properties and scores 3 on the ACB scale and the AEC scale. It is a medication associated with an increased risk of cognitive impairment. Mrs. AT was willing to try to stop both medication; however, her GP agreed to stop the pregabalin and continue the amitriptyline. He was reluctant to stop both in case Mrs. AT’s pain had a nerve component.

**vi, vii, and viii.** Mrs. AT was prescribed a Clenil modulate inhaler (Beclometasone dipropionate), salbutamol CFC inhaler (propellant free), montelukast, and carbocisteine. There was no documented indication for these medications in Mrs. AT’s medical record. They appeared to have been started after treatment for an acute chest infection. Mrs. AT had multiple co-morbidities. Her breathlessness could have been of respiratory or cardiac origin. There was no record of any spirometry, BNP (B-type natriuretic peptide), or echocardiogram tests or results to confirm or refute the possible diagnosis. The GP agreed to stop the montelukast and carbocisteine, but wished to continue the inhalers. Mrs. AT’s inhaler technique was assessed, and she was asked to monitor if the inhalers relieved her breathlessness. If she found no benefit from the inhalers, then this was to be discussed further with her GP.

**ix.** Mrs. AT had been taking Forceval^®^ capsules for two years. She was now having difficulty swallowing them. This was a non-formulary drug, and should only be prescribed to prevent or treat specific deficiency states or where the diet is known to be inadequate. Mrs. AT had no indication for a multivitamin preparation to be prescribed. The use of vitamins as general ‘pick-me-ups’ is unproven. If a patient wishes to take a multivitamin preparation where there is no clinical indication, they should be advised to purchase an OTC (over the counter) once-a-day multivitamin and mineral preparation. Mrs. AT was happy to stop Forceval^®^ due to problems she experienced taking it. Her GP agreed to this.

**x.** Lidocaine patches were prescribed for Mrs. AT’s osteoarthritis pains in her knees. This was an unlicensed and a non-formulary indication. There is no evidence that lidocaine patches relieve osteoarthritis pain. This was discussed with Mrs. AT. She was willing to try stopping the lidocaine patch with the understanding that it could be restarted if her pain increased on stopping it.

Mrs. AT had osteoporosis and a history of falls. She had previously been prescribed alendronic acid 70 mg weekly and colecalciferol 800 units per day, but had only taken these for a few months. Mrs. AT did not know why these medicines had been stopped. She said she had never taken a calcium supplement. Notably, her calcium intake was assessed, and was >700mg/ day. Mrs. AT agreed to restart the alendronic acid and colecalciferol. This was discussed with her GP, and the medications were represcribed.

Mrs. AT had a documented history of coronary heart disease, but was not prescribed a statin. Her cholesterol level was 4.2mmol/L. The evidence for the benefits of statins decreases after the age of 85. Mrs. AT’s preference was to reduce her pill burden; hence, it was not considered appropriate to start a statin.

#### 3.1.3. Steps 5, 6, and 7: Agree Actions to Stop, Reduce Dose, Continue, or Start; Communicate Actions with All Relevant Parties; Monitor and Adjust Regularly

Having balanced the risk and benefits of all the medicines, the pharmacist discussed the proposed actions with the patient’s GP and agreed which actions would be implemented. The agreed actions were:

- Lansoprazole reduced from 30 mg twice a day to 15 mg twice a day
- Pregabalin stopped
- Montelukast stopped
- Carbocisteine stopped
- Forceval stopped
- Lidocaine patch stopped
- Alendronic acid 70 mg weekly restarted
- Colecalciferol 800 units daily restarted.

The following proposed actions were not agreed by Mrs. AT’s GP:

- Stopping nitrofurantoin
- Changing furosemide dose to once a day
- Stopping amitriptyline
- Discontinuing beclomethasone and salbutamol inhalers.

Details of the agreed actions were summarised in a discharge letter to the GP. Verbal and written explanation of the medication changes was given to the patient and her daughter. The patient received a telephone follow up after two weeks. Mrs. AT reported no adverse effects from stopping these medications or from restarting the alendronic acid and colecalciferol. She had no reflux symptoms and was agreeable to try reducing the lansoprazole to 15 mg once a day. Her pain had not increased since stopping the lidocaine patch and pregabalin. Mrs. AT had not suffered from any breathlessness after stopping the montelukast, and had not had to use her salbutamol inhaler. There had been no increase in sputum production or viscosity following discontinuation of the carbocisteine. Mrs. AT was delighted that her pill burden had been decreased. Her daughter said that the best part of her mother’s intermediate care admission was meeting the pharmacist and reducing the amount of medications she had to take.

### 3.2. Care Home

#### 3.2.1. Steps 1 and 2: Assess Patient; Define Context and Overall Goals

Mrs. HJ, an 87-year-old lady who was a permanent resident in a nursing home, was reviewed by the care home pharmacist. Her medical history documented that she had Alzheimer’s dementia, chronic kidney disease (stage not specified), osteoarthritis, previous TIAs (transient ischaemic attacks), previous CVA (cerebral vascular accident), and a right total knee replacement.

She used a rollator when walking.

Her blood pressure and pulse were measured monthly, and the results over the last three months are presented in Table 4 below.

Her height was 160 cm, she weighed 77.3 kg, and her body mass index (BMI) was 29.6 kg/m^2^. Her current estimated glomerular filtration rate (eGFR) was 34 mL/min/1.73 m^2^.

She had gradually become more confused since admission to the care home three years previously. She was unable to discuss her needs and concerns during the medication review due to cognitive impairment. Where people do not have capacity to discuss and make decisions about treatment, medicines would normally be reviewed with relatives or informal carers to gain insight into the person’s wishes and beliefs. As Mrs. HJ did not have any family, her medicines were reviewed with care home staff. No issues were identified with medicines adherence. Mrs. HJ was always compliant with administered medication and had no swallowing difficulties.

Nursing staff reported that pain management for pain in both her knees was important to Mrs. HJ. She had one fall in the past year at her bedside. She was assessed as being moderately to severely frail using the Rockwood scale, with a score of 6–7, requiring assistance for some activities and completely dependent for some personal care.

Her medications were as follows:

- Donepezil 10 mg once daily at night
- Oxycodone MR (Longtec^®^) 5 mg in the morning, 10 mg at night
- Paracetamol 1 g four times daily
- Pregabalin 75 mg in morning
- Furosemide 40 mg in the morning
- Clopidogrel 75 mg once daily
- Omeprazole 20 mg once daily
- Atorvastatin 10 mg at night
- Ferrous fumarate 305 mg once daily
- Diclofenac gel 1% apply three times daily when required for knee pain
- Macrogol sachet one sachet twice daily when required

She had no allergies.

The anticholinergic effect on cognition (AEC) score was 0 and the anticholinergic burden (ACB) score was 1 for all her medications [34,35].

#### 3.2.2. Steps 3 and 4: Identify Medicines with Potential Risks; Assess Risks and Benefits in Context of Individual Patient

The pharmacist had undertaken a pre-person review, and identified the following issues to discuss with care home staff during the review:

- Alzheimer’s treatment
- Pain management
- Diuretic treatment
- CVA prevention–medication interaction between clopidogrel and omeprazole
- Anticholinergic effect of cognition (AEC) scale and anticholinergic burden (ACB)
- Ferrous fumarate
- Use of statins
- Constipation

**i.** Mrs. HJ was prescribed donepezil for her Alzheimer’s disease. The dose had appropriately been titrated to the maximum dose of 10 mg daily, and was being administered at night as recommended. Donepezil can commonly cause (>1 in 100 to <1 in 10) side effects of abnormal dreams, nightmares, and hallucinations or insomnia [40]: however, Mrs. HJ had not experienced any of these issues. Donepezil was to be reviewed again if Mrs. HJ progressed to severe frailty with consideration of the addition of memantine if behavioural problems occurred. Care home staff were advised to be vigilant for delirium if Mrs. HJ’s frailty progressed.

**ii.** Mrs. HJ’S main concern was her pain management. The therapeutic goal for her was to effectively manage her pain to optimise her quality of life. The National Institute of Health and Care Excellence (NICE) Dementia NG97 [41] recommends using a structured observational pain assessment tool with a self-reported pain level for people living with moderate to severe dementia who are unable to self-report pain; however, this patient was able to verbalise her pain levels. The guidance recommends a stepwise treatment protocol that balances pain management and potential adverse events.

The care home staff explained that Mrs. HJ’s pain was previously uncontrolled, but was now managed using Longtec^®^ (oxycodone) 5 mg in the morning and 10 mg at night, pregabalin 75 mg morning, paracetamol 1 g four times daily, and diclofenac 1% gel. Care home staff perceived that Mrs. HJ had become more confused since starting pregabalin three months ago. It was agreed to taper the pregabalin dose down and eventually stop it. An analgesia management plan was commenced to reduce the pregabalin dose to 50 mg daily and then reduce it further in one month to 25 mg daily if Mrs. HJ was stable, and then stop it after a further month. The care home staff continued to repeat pain assessments. If Mrs. HJ’s dementia progressed, this would be even more important to ensure that her pain was being assessed appropriately, as she may not be able to communicate her pain verbally. Care home staff were advised to be vigilant for issues such as delirium, which may indicate uncontrolled pain.

**iii.** There was no indication for ongoing furosemide treatment. Mrs. HJ’s recent blood pressure readings previously described in Table 4 were low 100/70 mmHg (Sept 2018), 98/60 mmHg (August 2018), and 100/60 mmHg (July 2018). Taking account all of the information, furosemide had the potential to lower Mrs. HJ’s blood pressure with no treatment benefit. Mrs. HJ’s falls risk was medium; she had a falls history of one fall by her bedside, she mobilised with a rollator, and with ongoing low blood pressure, she was at risk of another fall. Furosemide has an ACB score of 1.

The pharmacist liaised with Mrs. HJ’s GP regarding the discontinuation of furosemide. The GP agreed to stop the furosemide with the ongoing monthly monitoring of blood pressure, as well as monitoring ankle oedema. Care home staff were advised to report any ankle swelling to the GP practice.

It is important to take account of Mrs. HJ’s moderate to severe frailty level, as the European Society of Cardiology/European Society of Hypertension (ESC/ESH) hypertension guidelines 2018 [42] indicate it is appropriate to relax the BP target to <160/90 mmHg with no postural blood pressure drop.

**iv.** Mrs. HJ was prescribed clopidogrel as secondary prevention following a previous stroke. This treatment is in line with NICE Clinical Knowledge Summary (CKS) Stroke and TIA [43], which states that antiplatelet therapy is initiated by secondary care on diagnosis of ischaemic stroke or TIA without paroxysmal or permanent atrial fibrillation for long-term vascular prevention. Standard licensed treatment in ischaemia stroke is 75 mg daily. Omeprazole had been prescribed long term as gastrointestinal protection alongside the antiplatelet treatment.

There is conflicting evidence regarding the interaction between clopidogrel and PPIs. The British National Formulary (BNF) [36] states that “omeprazole is predicted to decrease the efficacy of clopidogrel” and the manufacturer advises avoiding this combination. PPIs can also increase the risk of fractures, particularly when used at high doses for over a year in adults over 60 years of age. It was agreed with Mrs. HJ’s GP to stop the omeprazole and to commence lansoprazole 15 mg daily. Care home staff were asked to monitor if Mrs. HJ developed any gastrointestinal symptoms.

**v.** Anticholinergic effect on cognition (AEC) scale and anticholinergic burden (ACB) scale

The anticholinergic effect on cognition score was calculated for this patient. Medication was rated using the Medichec tool [35], and the AEC score was calculated to be 0. The ACB score was calculated to be 1 for furosemide [34]. The furosemide was discontinued, reducing the ACB score to 0. At the end of the review, Mrs. HJ was not prescribed any medicines that could cause a concerning level of anticholinergic effect on this patient’s cognition. This was to be reviewed if there were any changes to Mrs. HJ’s medication.

**vi.** Ferrous Fumarate

This lady had been on ferrous fumarate treatment for anaemia long term. It was agreed with her GP to stop the oral iron, as her iron profile was satisfactory.

**vii.** Atorvastatin

This patient was currently prescribed atorvastatin 10 mg at night. NICE TIA management (2017) [43] advises prescribing a high-intensity statin such as atorvastatin 20–80mg to all patients post-TIA to promote a >40% reduction in non-HDL (high-density lipoprotein) cholesterol. The date of commencement of atorvastatin 10 mg, which is a moderate-intensity statin, is unclear. This did not allow a calculation of percentage reduction in non-HDL cholesterol as recommended, but it was important to note that Mrs. HJ’s lipid profile was within a safe range. Cholesterol = 3.1 (4 mmol/L or less for those at high risk, National Health Service (NHS) conditions website), LDL (low-density lipoprotein) = 1.1 (2 mmol/L or less for those at high risk) and HDL = 1.4 (the ideal level of HDL is above 1 mmol/L).

There was complexity in Mrs. HJ’S case, as she was living with frailty and multiple co-morbidities. Taking account of her frailty score, the long-term benefit of atorvastatin against the risk of side effects needed to be considered. Following discussion with her GP, it was decided that the atorvastatin dose would be maintained at 10 mg, and would be reviewed following any change in Mrs. HJ’s condition or frailty score, as there is no added value of a statin once frailty progresses to severe frailty and end of life approaches (Rockwood score 7–9).

**viii**. Macrogol

Mrs. HJ was currently taking a macrogol, which is an osmotic laxative, on a when-required basis to treat constipation. Her constipation was managed effectively on this one agent, and the treatment is in line with NICE CKS Constipation in Adults (2017) [44]. Therefore, no other medication was required, and continuation of this medication was recommended.

#### 3.2.3. Steps 5, 6 and 7: Agree Actions to Stop, Reduce Dose, Continue or Start; Communicate Actions with all Relevant Parties; Monitor and Adjust Regularly

The pharmacist’s review optimised Mrs. HJ’s medication to improve the outcomes attained and minimise adverse effects from her medication. A reduction in inappropriate polypharmacy had a positive impact on Mrs. HJ, minimising her pill burden and managing her long-term conditions more effectively.

The pharmacist discussed the proposed actions with the patient’s GP and agreed which actions would be implemented. The agreed actions following the pharmacist-led medication review were:

- Alzheimer’s management: donepezil continued
- Falls risk and anticholinergic burden reduced by discontinuation of furosemide
- Reduction in pregabalin dose from 75 mg to 50 mg once a day for one month, to be further tapered to 25 mg once a day, and then stopped after a further month if pain was controlled
- Longtec, paracetamol, and diclofenac gel continued with unchanged dose to manage pain
- Ferrous fumarate stopped
- Cardiovascular risk reduced by changing the choice of the proton pump inhibitor from omeprazole to lansoprazole
- Atorvastatin dose not increased
- Constipation managed with when required macrogol.

Details of the agreed actions were summarised in a letter to the GP and documented in the patient’s care plan. The patient was followed up on the next two subsequent visits to the nursing home. Mrs. HJ did not suffer any adverse effects or deterioration in her long-term conditions from the changes to her medication. The pregabalin continued to be tapered, and was stopped after two months.

## 4. Summary

This article discusses how person-centred medicines optimisation for older people, including the process of deprescribing, can be conducted in clinical practice, sharing techniques, frameworks, and tools that can be used to deprescribe safely whilst placing the person’s views, values, and beliefs about their medicines at the heart of any deprescribing discussions. The authors believe that delivering deprescribing consultations in this manner is more effective, as the person is integral to the deprescribing decision-making process.

## Figures and Tables

**Table 1:** Simple approach to classifying medication.

Medication Class	Questions to Consider	Comments
Symptom control	◾ Are symptoms currently being controlled? ◾ How can we be sure that this is due to continued use of the medication? ◾ Could the condition have been self-limiting?	◾ Need to challenge the status quo. ◾ Medication could be delivering very little benefit whilst still exposing the person to potential harm. ◾ Only way of knowing completely is by taking a medication holiday, monitoring, and reviewing
Risk reduction	◾ Are these medications still appropriate and delivering the same intended benefit as they were originally prescribed for in light of the person’s current circumstances?	◾ Need to perform a new risk assessment benefit each time. ◾ Risk benefits changes over time. ◾ People’s goals also change.

**Table 2:** The four Es—explore, educate, empower, and enable (© Nina Barnett).

◾ Explore what the person wants to know and follow their agenda ◾ Educate them on what they want to know ◾ Empower persons to take responsibility for medicines taking ◾ Enable behavioural change in order for the person to achieve their aims

**Table 3:** The Capability, Opportunity, and Motivation (COM-B) model of behaviour.

For an Individual to Undertake a Behaviour They Must:	Problems May Include
**1. Be Capable of doing it**	◾ Knowledge of disease ◾ Understanding of medicines ◾ Cognitive impairment/memory ◾ Ability to use devices
**2. Have Opportunity to do it**	◾ Challenge of high medicine burden or complex regimens ◾ Social support—Knowledge/Beliefs/support of partners and carers ◾ Trust/faith/belief in healthcare professionals
**3. Be Motivated to do it**	◾ Perceived need for treatment (low when well) ◾ Perception of disease ◾ Concerns about side effects ◾ Lack of confidence in adherence (habit of medicine taking) ◾ Mental health issues

**Table 4:** Monthly blood pressure (BP) and pulse readings for Mrs. HJ.

Month (2018)	July	August	September
BP (mmHg)	100/60	98/60	110/70
Pulse (bpm)	71	67	76
